# Nucleotide metabolism reprogramming in obesity-associated cardiovascular inflammation: a new perspective

**DOI:** 10.3389/fimmu.2026.1829718

**Published:** 2026-04-24

**Authors:** Taoming Qian, Meijun Zhang, Yuhan Liu, Donghao Guo, Juan Jin

**Affiliations:** 1 Graduate School, Heilongjiang University of Chinese Medicine, Harbin, China; 2 Department of Peripheral Vascular Diseases II, The First Affiliated Hospital of Heilongjiang University of Chinese Medicine, Harbin, China; 3 Department of Cardiovascular Diseases I, The First Affiliated Hospital of Heilongjiang University of Chinese Medicine, Harbin, China

**Keywords:** atherosclerosis, cardiovascular inflammation, dNTP pools, immunometabolism, macrophage reprogramming, mitochondrial DNA, NLRP3 inflammasome, nucleotide metabolism

## Abstract

Obesity drives cardiovascular disease primarily through chronic meta-inflammation, yet the precise molecular convergence linking nutrient excess to sustained NLRP3 inflammasome activation in macrophages has remained unclear. Obesity induces inhibitory phosphorylation of SAMHD1, resulting in cytosolic dNTP accumulation, mitochondrial import through SLC25 transporters, uncontrolled mtDNA synthesis and oxidation, and consequent NLRP3 hyperactivation. This SAMHD1–dNTP–mtDNA–NLRP3 axis is supported by emerging evidence as a potential upstream checkpoint that may set macrophage inflammatory tone and may contribute to three major manifestations of obesity-associated cardiovascular pathology: accelerated atherosclerosis, diastolic dysfunction in obesity cardiomyopathy, and exaggerated ischemia–reperfusion injury. Integration of human macrophage data, atherosclerotic plaque biology, and mitochondrial transport mechanisms reveals actionable therapeutic nodes at selective SLC25 inhibition, SAMHD1-preserving kinase modulation, and mitochondria-targeted antioxidants that act at the metabolic initiation phase rather than downstream effector cascades. Nucleotide metabolism reprogramming thereby provides a plausible framework that may help address a long-standing mechanistic gap in immunometabolism and opens a new class of precision interventions that could address the root cause of obesity-driven cardiovascular risk.

## Introduction

Obesity has emerged as the dominant modifiable risk factor for cardiovascular disease globally, with epidemiological studies demonstrating that each 5 kg/m² increase in body-mass index confers an approximately 30% higher risk of coronary heart disease (1). While the association is well-established, the mechanistic basis lies in obesity-induced chronic low-grade inflammation—a persistent state of immune activation that promotes endothelial dysfunction, accelerates atherosclerotic plaque progression, and impairs myocardial metabolic adaptation (2). The central scientific question is not whether inflammation occurs, but rather how nutrient excess is sensed and transduced into sustained immune activation.

Despite two decades of research establishing the concept of “meta-inflammation,” the precise molecular mechanisms by which nutritional excess translates into sustained immune activation have remained incompletely understood (2, 3). The NLRP3 inflammasome has emerged as a central executor of sterile inflammation in cardiovascular pathology, with its activation leading to IL-1β and IL-18 production that drives vascular inflammation, plaque instability, and myocardial dysfunction (4, 5). Cholesterol crystals, oxidized lipoproteins, and saturated fatty acids—all enriched in the obese state—have been identified as potent NLRP3 agonists (4, 6). However, how these diverse metabolic stressors converge upon a common inflammatory pathway has been a critical knowledge gap. This gap is fundamental: without understanding the convergence point, therapeutic development remains empirical rather than mechanism-based.

A recent study has illuminated one unexpected convergence point: nucleotide metabolism (7). Liu, Zhong and colleagues demonstrated that obesity promotes inhibitory phosphorylation of SAMHD1, a conserved dNTP hydrolase, leading to cytosolic dNTP accumulation in macrophages. These excess nucleotides are transported into mitochondria, driving uncontrolled mtDNA synthesis, oxidative damage, and subsequent NLRP3 hyperactivation (7). This work reveals that the metabolic rewiring associated with obesity directly interfaces with innate immune machinery at the level of nucleotide pools—a connection previously unappreciated. The critical insight is that nucleotide metabolism is not merely a housekeeping function but an active participant in setting the inflammatory threshold of macrophages. Although cholesterol crystals, oxidized lipoproteins, and saturated fatty acids have been established as distinct NLRP3 agonists, emerging evidence suggests that these diverse metabolic stressors may converge upstream on SAMHD1 inactivation under obesogenic conditions. For instance, saturated fatty acids such as palmitate potently activate mTORC1-S6K signaling (8), and mTORC1 is a key kinase implicated in SAMHD1 Thr592 phosphorylation. Oxidized LDL and cholesterol crystals induce lysosomal damage and oxidative stress, which can further engage MAPK family kinases to promote inhibitory SAMHD1 phosphorylation. Liu et al. (7) directly demonstrated elevated SAMHD1 phosphorylation, dNTP accumulation, and NLRP3 hyperactivation in macrophages from obese individuals, with this effect conserved across zebrafish, mouse, and human models. These findings indicate that rather than acting in parallel, classical NLRP3 agonists in the obese state may functionally converge through nucleotide metabolism reprogramming, positioning the SAMHD1–dNTP–mtDNA–NLRP3 axis as a proposed unifying upstream checkpoint supported by emerging evidence.

The present perspective builds upon this foundational observation to examine broader implications for cardiovascular pathophysiology. Rather than focusing on the specific experimental findings, we consider how altered nucleotide metabolism may reshape our understanding of obesity-associated cardiovascular inflammation and identify new therapeutic opportunities. The central thesis, as proposed here, is that nucleotide metabolism may represent an upstream checkpoint influencing macrophage inflammatory setpoints, with direct consequences for atherosclerosis, myocardial dysfunction, and ischemia–reperfusion injury.

## Nucleotide metabolism as an immunometabolic hub

Nucleotide metabolism has traditionally been viewed through the lens of cell proliferation and DNA synthesis, with limited consideration of its immunoregulatory functions. This view is no longer tenable. The recognition that dNTP pools are tightly regulated and that their dysregulation triggers inflammatory responses represents a conceptual advance with broad implications.

### SAMHD1: beyond antiviral restriction

SAMHD1 functions as a triphosphohydrolase that cleaves dNTPs into constituent nucleosides and inorganic phosphate, maintaining intracellular nucleotide homeostasis (9). Its well-characterized role in restricting HIV-1 replication by depleting substrates required for reverse transcription has dominated the literature (10). However, emerging evidence suggests broader functions in immune regulation. SAMHD1 deficiency in humans causes Aicardi–Goutières syndrome, an autoinflammatory disorder characterized by type I interferon overproduction, indicating its role in preventing inappropriate nucleic acid sensing (11). This connection between a nucleotide-metabolizing enzyme and spontaneous autoinflammation is instructive: it demonstrates that the immune system constantly monitors nucleotide pools as a surrogate for cellular homeostasis.

The convergence of antiviral restriction, autoinflammation prevention, and metabolic regulation positions SAMHD1 as a molecular node integrating diverse cellular inputs. A key mechanistic question is how SAMHD1 activity is regulated in response to metabolic stress. Its sensitivity to post-translational modification—particularly phosphorylation at Thr592—allows rapid adjustment of dNTP pools in response to cell cycle status, metabolic conditions, and inflammatory signals (12). This regulatory complexity suggests that SAMHD1 functions as a rheostat rather than a simple on-off switch, fine-tuning nucleotide availability according to cellular context. The phosphorylation status of SAMHD1 may therefore serve as a molecular readout of metabolic stress, linking systemic obesity to cellular inflammatory potential. Given that plaque macrophages are largely non-proliferative, the classical CDK1/2 axis is unlikely to operate here. We hypothesize that stress-responsive kinases, such as members of the MAPK family or mTOR-associated kinases (S6K), may usurp this function under obesogenic conditions, representing a key divergence from the canonical cell-cycle linked regulation.

### The dNTP–mitochondria–inflammation axis

Mitochondria contain their own genome, which requires dNTPs for replication. Unlike nuclear DNA synthesis, which is tightly coupled to cell division, mtDNA turnover occurs continuously in post-mitotic cells, including macrophages (13). Mitochondria import cytosolic dNTPs through specific nucleotide transporters, primarily members of the SLC25 family such as SLC25A33 and SLC25A36, though the full complement of transporters responsible for dNTP import remains incompletely characterized (14).

The recognition that expanded cytosolic dNTP pools drive mtDNA synthesis and oxidation establishes a direct metabolic link to inflammasome activation. Oxidized mtDNA functions as a damage-associated molecular pattern recognized by NLRP3, triggering caspase-1 activation and IL-1β maturation (15). This obesity-driven pathway bypasses the canonical CMPK2-dependent mitochondrial dNTP salvage mechanism previously shown to be required for controlled NLRP3 activation under normal conditions (16). A critical mechanistic insight is that this pathway operates independently of canonical inflammasome priming signals, suggesting that nucleotide pool expansion alone may be sufficient to lower the threshold for inflammatory activation. This has profound implications: it implies that metabolic state directly sets the baseline inflammatory tone of macrophages.

**Figure 1:**
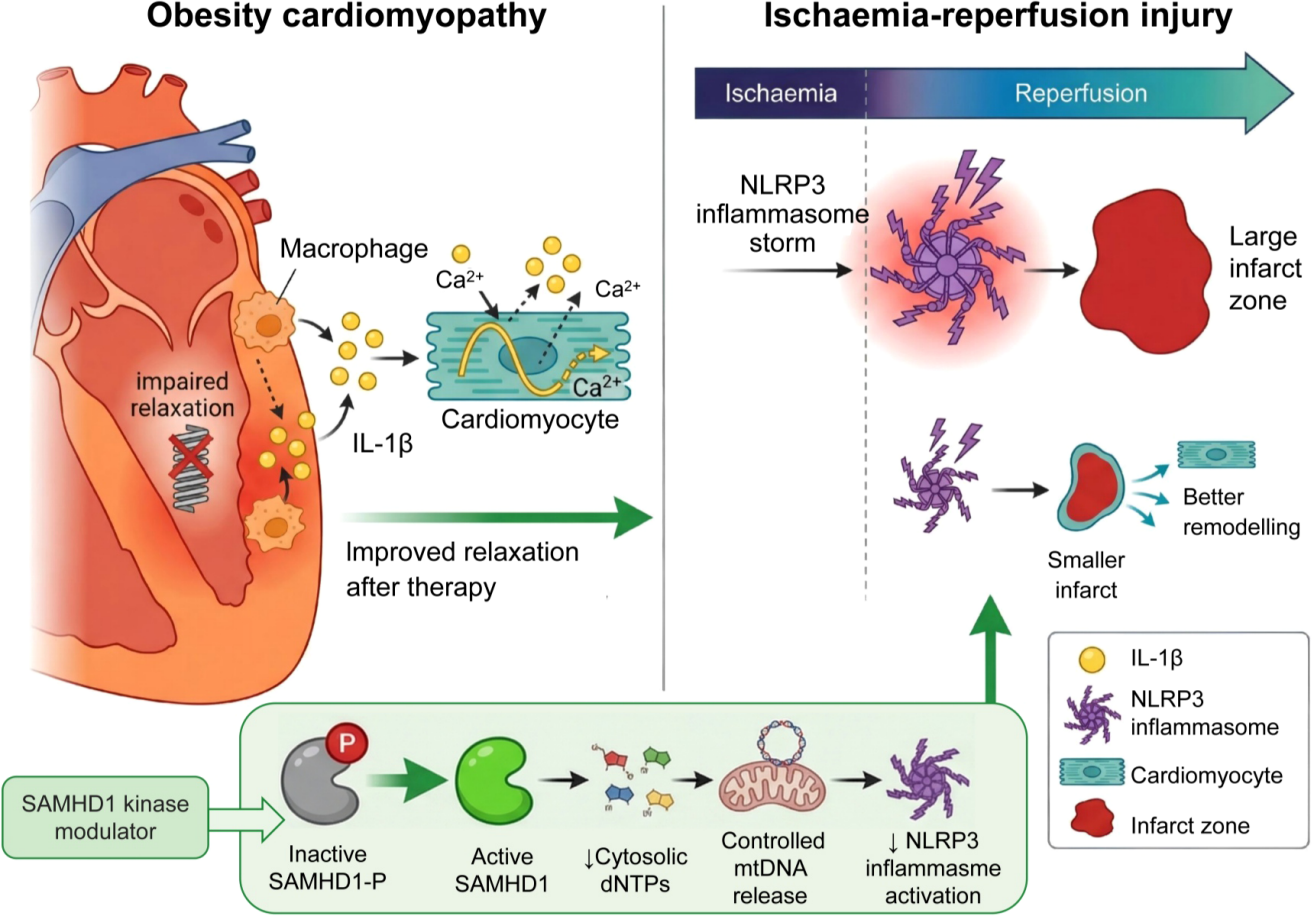
Schematic representation of the proposed SAMHD1–dNTP–SLC25–mtDNA–NLRP3 axis in obesity-associated macrophage inflammation and cardiovascular disease. In the proposed model, obesity induces inhibitory phosphorylation of SAMHD1 (shown as a grayed-out hydrolase with red “P”), leading to cytosolic accumulation of deoxyribonucleotide triphosphates (dNTPs, depicted as overflowing A/T/C/G icons). Excess dNTPs are transported into mitochondria via SLC25A33/SLC25A36 transporters (green channel proteins), resulting in excessive mtDNA synthesis (increasing green mtDNA circles). Subsequent mtDNA oxidation and release into the cytosol (red-damaged circles) activates the NLRP3 inflammasome complex (NLRP3, ASC and caspase-1 assembly with lightning bolt), triggering maturation and release of IL-1β and IL-18 (flame icon and cytokine molecules) and sustained sterile inflammation. The top-right inset compares the lean state (balanced dNTP pools and controlled mtDNA turnover, blue/green tones) with the obese state (hyperactivation vicious cycle indicated by dashed red feedback arrow). Key molecular components are summarized in the right-side legend panel: SAMHD1, dNTP, SLC25 transporters, mtDNA, NLRP3 inflammasome, and IL-1β/IL-18.

For cardiovascular biology, this mechanism has particular relevance. Macrophages in atherosclerotic plaques exist in a metabolically stressed environment characterized by hypoxia, oxidized lipids, and altered substrate availability (17). If SAMHD1 activity is compromised in these cells, sustained dNTP accumulation could drive continuous mtDNA turnover and oxidative damage, creating a feed-forward loop of inflammatory activation independent of classical danger signals. The key unresolved question is whether this mechanism operates continuously in plaque macrophages or is triggered intermittently by metabolic fluctuations. As illustrated in **Figure 1**, obesity compromises SAMHD1 activity, resulting in cytosolic dNTP overflow that is imported into mitochondria and drives excessive mtDNA synthesis and oxidation, thereby activating the NLRP3 inflammasome in macrophages. This schematic highlights the proposed feed-forward loop in the obese state versus controlled homeostasis in the lean state, providing a visual framework for the axis discussed above.

To facilitate clear distinction between established findings and hypothesis-driven extrapolations within the proposed SAMHD1–dNTP–mtDNA–NLRP3 axis, the key mechanistic steps, supporting evidence, limitations, and translational implications are systematically summarized in **Table 1**.

**Table 1:** Summary of the proposed SAMHD1–dNTP–mtDNA–NLRP3 axis in obesity-associated cardiovascular inflammation.

Mechanistic step	Supporting evidence	Evidence type	Relevance to obesity-associated cardiovascular disease	Current limitations	Potential translational implications
Obesity-induced inhibitory phosphorylation of SAMHD1 in macrophages	Elevated p-SAMHD1, dNTP accumulation, and NLRP3 hyperactivation in obese macrophages; linked to mTORC1-S6K by palmitate	*In vitro*, animal (mouse, zebrafish), human macrophages	Primes macrophages for meta-inflammation in obese metabolic milieu, amplifying plaque and myocardial inflammation	Kinase identity in non-proliferative macrophages remains unknown	Kinase modulators to preserve SAMHD1 as upstream metabolic intervention
Cytosolic dNTP accumulation	SAMHD1 inactivation leads to expanded dNTP pools (7)	*In vitro*, animal, human	Fuels continuous nucleotide supply in stressed plaque and cardiac macrophages	Direct dNTP quantification in human plaques limited	Potential biomarker for pathway activation in obese CVD patients
Mitochondrial dNTP import via SLC25 transporters	SLC25A33/A36 candidates mediate import; excess drives mtDNA synthesis,	Primarily *in vitro*; proposed *in vivo*	Sustains mtDNA turnover in hypoxic/obese cardiovascular tissues	Transporter identity and obesity-specific regulation incomplete	Selective SLC25 inhibitors during metabolic stress
Excessive mtDNA synthesis and oxidation	Oxidized mtDNA as NLRP3 ligand; obesity-driven via dNTP excess	*In vitro*, animal models	Triggers sterile inflammation in atherosclerosis, cardiomyopathy, and ischaemia–reperfusion injury	Human correlative data predominate; causality unproven	Mitochondria-targeted antioxidants (e.g., MitoQ) to limit oxidative damage
NLRP3 inflammasome activation and IL-1β/IL-18 release	Oxidized mtDNA activates NLRP3 leading to cytokine maturation	*In vitro*, animal, human association	Drives accelerated plaque progression, diastolic dysfunction, and exaggerated reperfusion injury	Pathway contribution vs. classical agonists not fully dissected in multi-disease models	New class of upstream therapies complementing downstream IL-1β blockade

The table distinguishes direct experimental evidence from hypothesis-driven mechanistic extrapolations specific to obesity-associated cardiovascular disease (labeled as “proposed”). Evidence types are classified strictly as *in vitro*, animal models, or human data; current limitations explicitly note gaps in causality or clinical validation; translational implications focus exclusively on potential upstream nodes supported by emerging data. All interpretations remain tentative pending rigorous *in vivo* causality studies and human translational confirmation.

## Cardiovascular consequences of metabolic–immune dysregulation

The conceptual framework linking nucleotide metabolism to inflammation has specific implications for cardiovascular disease that extend beyond general principles of immunometabolism. We now examine three domains where this framework generates testable hypotheses and may explain clinical observations.

### Atherosclerosis: a disease of metabolic inflammation

Atherosclerosis is now recognized as a chronic inflammatory disease of the arterial wall, with macrophages playing central roles in lipid handling, plaque progression, and complications. The discovery that cholesterol crystals activate NLRP3 provided a direct mechanism linking lipid deposition to inflammation. However, the nucleotide metabolism axis suggests additional layers of complexity.

Plaque macrophages exhibit profound metabolic reprogramming characterized by shifts in glucose utilization, fatty acid oxidation, and mitochondrial function (17). The hypoxic plaque microenvironment further stresses mitochondrial homeostasis, promoting mtDNA damage and release. Oxidized mtDNA serving as a potent NLRP3 ligand has been extensively validated in atherosclerosis, with recent reviews (18, 19) emphasizing its central role in obesity-exacerbated plaque inflammation and progression. If obesity-associated SAMHD1 inactivation primes macrophages with expanded dNTP pools, these cells may be predisposed to exaggerated inflammatory responses upon encountering plaque-derived stimuli. This raises the testable hypothesis that obese individuals exhibit more extensive plaque inflammation and higher rates of plaque rupture (20). The hypothesis is testable: plaque macrophages from obese donors should show elevated SAMHD1 phosphorylation, expanded dNTP pools, and increased mtDNA oxidation compared to lean controls. This pathway is embedded in the inflammasome-mediated cardiovascular-metabolic-immune comorbidity networks that amplify obesity-driven atherosclerosis and myocardial dysfunction (21).

The nucleotide metabolism axis may also intersect with other plaque characteristics. Intraplaque hemorrhage exposes macrophages to erythrocyte-derived nucleotides, potentially amplifying dNTP pools through salvage pathways (22). Necrotic core formation, a key determinant of plaque vulnerability, is driven by macrophage apoptosis and defective efferocytosis—processes influenced by NLRP3 activation (23, 24). Whether dNTP-driven inflammation contributes to these features warrants investigation. A key question is whether interventions that normalize nucleotide pools can stabilize existing plaques.

### Myocardial dysfunction in obesity

Obesity-associated cardiomyopathy manifests as diastolic dysfunction progressing to heart failure with preserved ejection fraction, a condition with limited therapeutic options (25). Myocardial inflammation, driven by infiltrating macrophages and resident cardiac immune cells, contributes to this pathology (26). The mechanistic link between obesity and myocardial inflammation, however, remains poorly defined.

The nucleotide metabolism framework suggests several mechanistic possibilities. Cardiac macrophages exhibiting SAMHD1 dysfunction may produce IL-1β that directly impairs cardiomyocyte calcium handling and insulin sensitivity (27). IL-1β suppresses cardiomyocyte contractility through effects on L-type calcium channels and sarcoplasmic reticulum calcium release, contributing to diastolic dysfunction (28). Additionally, IL-1β impairs insulin signaling in cardiomyocytes by inhibiting IRS-1 phosphorylation and GLUT4 translocation, promoting myocardial insulin resistance. The critical question is whether IL-1β derived from nucleotide-primed macrophages is quantitatively sufficient to produce these effects *in vivo*. While IL-1β from nucleotide-primed macrophages likely contributes to cardiomyocyte dysfunction, it probably acts in concert with other obesity-driven factors such as lipotoxicity and neurohormonal activation. The relative contribution of the SAMHD1 pathway versus these established mechanisms remains a key unknown.

Beyond macrophages, cardiomyocytes themselves contain abundant mitochondria and may be subject to similar nucleotide-mediated inflammatory mechanisms. Whether cardiomyocytes express functional NLRP3 inflammasomes remains debated, but they certainly release mtDNA upon stress, which can activate neighboring macrophages (29). The potential for cross-talk between metabolically stressed cardiomyocytes and nucleotide-primed macrophages represents an unexplored dimension of obesity-associated myocardial dysfunction. This paracrine loop could amplify local inflammation independently of circulating factors.

### Ischemia–reperfusion injury

Acute myocardial infarction triggers sterile inflammation that contributes to reperfusion injury and adverse ventricular remodeling. The magnitude of this inflammatory response correlates with infarct size and long-term outcomes. Obese patients experience worse outcomes after myocardial infarction, even with optimal reperfusion therapy. The explanation for this observation remains incomplete.

If obesity-associated SAMHD1 inactivation primes circulating monocytes and cardiac macrophages with expanded dNTP pools, these cells may mount exaggerated inflammatory responses upon encountering ischemic tissues. Reperfusion itself generates mitochondrial reactive oxygen species that could further amplify mtDNA oxidation, creating a vicious cycle (30). This predicts that the inflammatory response to infarction should be quantitatively greater in obese individuals, and that this difference should be attributable to the nucleotide–mitochondrial axis. Understanding whether nucleotide metabolism contributes to the “obesity paradox” in acute coronary syndromes—where obese patients sometimes show better short-term outcomes despite worse long-term prognosis—may reveal important insights into inflammatory regulation during acute stress (31, 32). We speculate that the acute protective effects often associated with obesity may involve metabolic pathways distinct from the SAMHD1-driven chronic inflammation, or that the nucleotide-mitochondrial axis is temporarily suppressed during the acute phase of ischemia, a hypothesis warranting further investigation.

## Therapeutic opportunities: targeting nucleotide metabolism for cardiovascular protection

The recognition that nucleotide metabolism directly interfaces with inflammatory activation opens multiple therapeutic avenues distinct from conventional anti-inflammatory strategies. The key principle is intervention at an upstream metabolic checkpoint rather than downstream inflammatory cascades.

### Limitations of current approaches

IL-1β blockade with canakinumab reduced recurrent cardiovascular events in the CANTOS trial but with modest effect size and increased infection risk (33). Direct NLRP3 inhibitors are under development but act after inflammatory cascades is initiated (34). These approaches, while validating the importance of IL-1β in atherosclerosis, highlight the need for interventions that prevent inflammation rather than suppressing established responses. The conceptual limitation is that they target the effector phase; the ideal intervention would target the initiation phase.

### A candidate upstream node: obesity-driven SAMHD1 phosphorylation

Although mitochondrial dNTP import represents an attractive downstream node, obesity-induced inhibitory phosphorylation of SAMHD1 is proposed as a potentially actionable node in this pathway. This phosphorylation, likely mediated by stress-responsive kinases such as mTOR/S6K or MAPK family members dysregulated in obesity, leads to dNTP accumulation at its source. Targeting the responsible kinase(s) to preserve SAMHD1 activity offers the most direct strategy to interrupt the axis at its metabolic origin. Identifying these kinases in terminally differentiated macrophages is therefore the highest-priority translational goal.

### Mitochondrial nucleotide transport as a secondary node

Blocking excessive mitochondrial dNTP import can abrogate NLRP3 hyperactivation when cytosolic pools are expanded, creating a therapeutic window during metabolic stress. While candidate transporters include SLC25A33 and SLC25A36 (35), their definitive identification and obesity-specific regulation remain to be fully elucidated. However, transporter inhibition should be considered complementary to upstream SAMHD1-preserving strategies rather than the primary target.

### SAMHD1 modulation

Strategies to preserve SAMHD1 function in obesity could prevent dNTP accumulation at its source. Identifying the kinase(s) responsible for obesity-associated SAMHD1 phosphorylation would enable more targeted intervention. The identity of this kinase is a critical gap. Potential candidates include CDK1, CDK2, and members of the mTOR signaling network, all of which are dysregulated in obesity (8). However, complete SAMHD1 activation risks excessive dNTP depletion with potential consequences for lymphocyte proliferation and antiviral immunity, suggesting that partial modulation or tissue-specific approaches may be required. The challenge is to achieve the right degree of modulation—enough to prevent pathological dNTP accumulation but not so much as to compromise essential functions.

### Mitochondria-targeted antioxidants

Because oxidized mtDNA is the proximate NLRP3 ligand, strategies that limit oxidative damage within mitochondria may complement nucleotide-directed approaches. MitoQ, a mitochondria-targeted antioxidant, improved endothelial function in older adults, suggesting cardiovascular benefits (36). Whether similar approaches limit NLRP3 activation in obesity remains to be tested. Combining mitochondrial antioxidants with nucleotide transport inhibitors could address both substrate supply and oxidative modification. The rationale for combination therapy is strong: reducing both the production of new mtDNA and its oxidative modification may achieve synergistic effects.

### Considerations for cardiovascular applications

**Figure 2:**
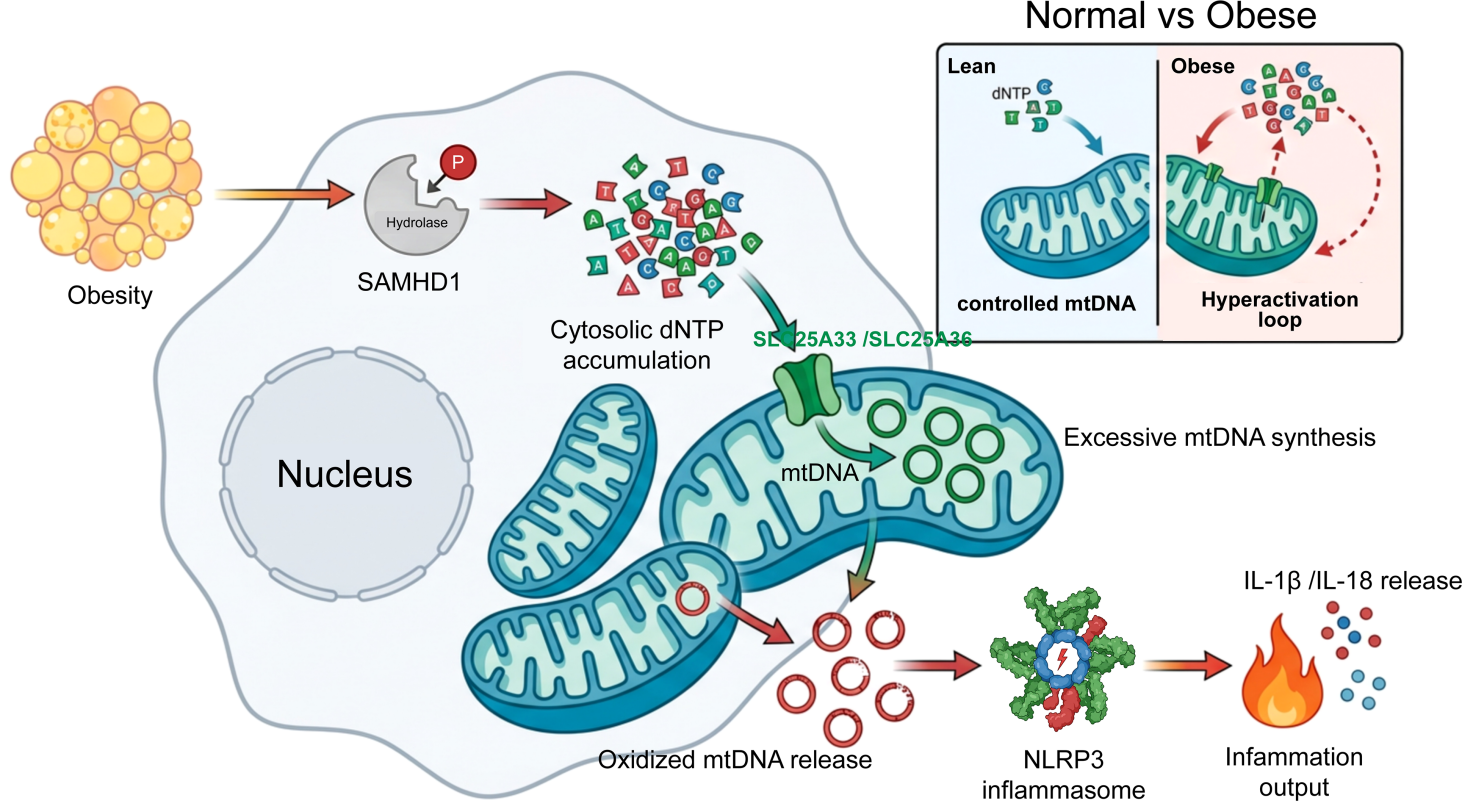
Resolution of obesity cardiomyopathy and ischemia–reperfusion injury through targeted modulation of the SAMHD1–dNTP–mtDNA–NLRP3 axis. In the left sub-panel (Obesity cardiomyopathy), macrophage-derived IL-1β impairs cardiomyocyte calcium handling (disrupted L-type Ca²^+^ channels and sarcoplasmic reticulum Ca²^+^ release), leading to diastolic dysfunction with impaired ventricular relaxation, while therapeutic intervention restores normal ventricular relaxation (green arrow); in the right sub-panel (Ischemia–reperfusion injury), the obese state exaggerates NLRP3 inflammasome activation during reperfusion, resulting in an inflammatory storm and enlarged infarct zone, with axis-targeted therapy attenuating inflammation, reducing infarct size, and improving ventricular remodeling (green arrows). A key upstream therapeutic node is illustrated in the bottom shared inset, where the SAMHD1 kinase modulator (green box/arrow) directly targets and prevents inhibitory phosphorylation of SAMHD1 at Thr592 (gray inactive SAMHD1 with red “P” → green active SAMHD1 without “P”), thereby restoring SAMHD1 dNTP hydrolase activity at the metabolic source, leading to decreased cytosolic dNTP pools (↓ dNTPs), controlled mitochondrial DNA synthesis and oxidation (controlled mtDNA), and reduced NLRP3 inflammasome activation (↓ NLRP3), with the green arrows extending from the modulator illustrating the downstream cascade interruption in both pathologies and highlighting intervention at the metabolic initiation phase rather than downstream effector suppression.

Any therapeutic strategy targeting nucleotide metabolism for cardiovascular indications must address several considerations. Chronic administration will be required for primary and secondary prevention, demanding exceptional safety profiles. Tissue selectivity may be important to avoid effects on hematopoietic progenitors or lymphocytes. The potential for sex-specific effects should be evaluated early, given sexual dimorphism in obesity-related cardiovascular risk (37). Finally, combination with existing cardiovascular therapies—statins, antihypertensives, antidiabetic agents—must be feasible without unexpected interactions. These are not afterthoughts but central considerations that will determine clinical translatability. **Figure 2** illustrates the potential resolution of two major obesity-associated cardiovascular pathologies—obesity cardiomyopathy and ischemia–reperfusion injury—through targeted modulation of the SAMHD1–dNTP–mtDNA–NLRP3 axis.

## Unresolved questions and future directions

Translating the nucleotide metabolism framework into cardiovascular therapeutics requires addressing fundamental questions that remain unanswered. We organize these around key scientific and translational gaps.

Cellular Specificity: Does SAMHD1 regulation differ among macrophage subsets in atherosclerotic plaques and cardiac tissue? Inflammatory subsets may prove particularly susceptible, necessitating subset-specific targeting strategies.

Tissue-Specific Drivers: Which obesity-associated signals—such as free fatty acids, adipokines, or oxidative stress—predominantly drive SAMHD1 inactivation in cardiovascular macrophages? Clarifying these will establish direct mechanistic links between adipose dysfunction and vascular inflammation.

Proximal Signaling Pathways: Identification of the specific kinases mediating obesity-driven SAMHD1 phosphorylation in terminally differentiated macrophages remains essential for enabling pharmacological preservation of SAMHD1 activity.

Mitochondrial Transporter Identification: Definitive molecular characterization of the SLC25 family transporters responsible for dNTP import under obesogenic conditions is a priority to support development of selective inhibitors.

*In Vivo* Validation: Genetic models, including SAMHD1 deficiency crossed with obese atherosclerotic backgrounds (e.g., ApoE^-^/^-^ mice), are required to establish causality in atherosclerosis progression and obesity cardiomyopathy.

Human Translation: Prospective clinical studies should determine whether SAMHD1 phosphorylation status in circulating monocytes or human plaques correlates with cardiovascular risk in obese individuals and predicts response to axis-targeted therapies.

## Conclusion

Obesity-associated cardiovascular disease remains a leading cause of morbidity and mortality worldwide, driven in large part by chronic inflammation that current therapies address incompletely. The recognition that nucleotide metabolism reprogramming directly interfaces with innate immune activation—exemplified by the SAMHD1–dNTP–mtDNA–NLRP3 axis—offers a new conceptual framework for understanding how metabolic dysregulation translates into cardiovascular risk. The central insight proposed here is that nucleotide pools may function as a rheostat for inflammatory setpoints.

This perspective has proposed that the nucleotide–mitochondrial inflammatory pathway may have specific implications for atherosclerosis, myocardial dysfunction, and ischemia–reperfusion injury that extend beyond general principles of immunometabolism. By proposing nucleotide metabolism as a potential upstream checkpoint that may control macrophage inflammatory setpoints, this framework identifies multiple potential therapeutic opportunities distinct from conventional anti-inflammatory approaches. A key conceptual distinction may lie between preventing inflammation at its metabolic source versus suppressing it after initiation. The critical path forward requires definitive identification of mitochondrial nucleotide transporters, elucidation of proximal signaling pathways controlling SAMHD1 activity, and rigorous preclinical validation in cardiovascular disease models. If successful, targeting nucleotide metabolism could yield a new class of cardiovascular drugs that may address the root cause of obesity-associated cardiovascular risk rather than suppressing its downstream manifestations. The question is no longer whether nucleotide metabolism regulates inflammation, but how we can therapeutically exploit this connection.

## Data Availability

The original contributions presented in the study are included in the article/supplementary material. Further inquiries can be directed to the corresponding authors.
